# Virulence Potential of Biofilm-Producing *Staphylococcus pseudintermedius*, *Staphylococcus aureus* and *Staphylococcus coagulans* Causing Skin Infections in Companion Animals

**DOI:** 10.3390/antibiotics11101339

**Published:** 2022-09-30

**Authors:** Mariana Andrade, Ketlyn Oliveira, Catarina Morais, Patrícia Abrantes, Constança Pomba, Adriana E. Rosato, Isabel Couto, Sofia Santos Costa

**Affiliations:** 1 Global Health and Tropical Medicine (GHTM), Instituto de Higiene e Medicina Tropical (IHMT), Universidade Nova de Lisboa (UNL), Rua da Junqueira 100, 1349-008 Lisboa, Portugal; 2 Centre of Interdisciplinary Research in Animal Health (CIISA), Faculty of Veterinary Medicine, University of Lisbon, Avenida da Universidade Técnica, 1300-477 Lisboa, Portugal; 3 GeneVet, Laboratório de Diagnóstico Molecular Veterinário, Rua Quinta da Nora Loja 3B, 2790-140 Carnaxide, Portugal; 4 Department of Pathology and Molecular Microbiology Diagnostics-Research, Riverside University Health System, Moreno Valley, CA 92555, USA; 5 School of Medicine, University of California Riverside, Riverside, CA 92521, USA

**Keywords:** *Staphylococcus pseudintermedius*, *Staphylococcus aureus*, *Staphylococcus coagulans*, virulence, biofilm, pyoderma, companion animals, *Galleria mellonella*

## Abstract

Coagulase-positive staphylococci (CoPS) account for most bacteria-related pyoderma in companion animals. Emergence of methicillin-resistant strains of *Staphylococcus pseudintermedius* (MRSP), *Staphylococcus aureus* (MRSA) or *Staphylococcus coagulans* (MRSC), often with multidrug-resistant (MDR) phenotypes, is a public health concern. The study collection comprised 237 staphylococci (*S. pseudintermedius* (*n* = 155), *S. aureus* (*n* = 55) and *S. coagulans* (*n* = 27)) collected from companion animals, previously characterized regarding resistance patterns and clonal lineages. Biofilm production was detected for 51.0% (79/155), 94.6% (52/55) and 88.9% (24/27) of the *S. pseudintermedius*, *S. aureus* and *S. coagulans,* respectively, and was a frequent trait of the predominant *S. pseudintermedius* and *S. aureus* clonal lineages. The production of biofilm varied with NaCl supplementation of the growth media. All *S. pseudintermedius* and *S. aureus* strains carried *icaADB*. Kaplan–Meier survival analysis of *Galleria mellonella* infected with different CoPS revealed a higher virulence potential of *S. aureus* when compared with other CoPS. Our study highlights a high frequency of biofilm production by prevalent antimicrobial-resistant clonal lineages of CoPS associated with animal pyoderma, potentially related with a higher virulence potential and persistent or recurrent infections.

## 1. Introduction

Skin infections, particularly pyoderma, are the main reason for antimicrobial prescription in companion animals [1]. Pyoderma is associated with pain, redness and inflammation of the skin [2]. Coagulase-positive staphylococci (CoPS) are amongst the main bacterial agents of these infections in companion animals [1,2,3]. In dogs, *Staphylococcus pseudintermedius* is responsible for over 90% of the cases, whereas *Staphylococcus aureus* and *Staphylococcus coagulans,* interchangeably recognized as the second or third pathogen most associated with these infections, account for up to 10% of pyoderma episodes [1,4]. The prevalence of pyoderma is lower in cats, ranging from 4% up to 20% [5,6], and is usually caused by *S. pseudintermedius*, *S. aureus* or coagulase-negative staphylococci [5]. In other companion animals, such as rabbits and horses, this infection is rare with only a few cases reported [7,8,9].

*S. pseudintermedius* was first described in 2005 by Devriese et al. and belongs to the *Staphylococcus intermedius* group (SIG), together with *Staphylococcus delphini* and *Staphylococcus cornubiensis* [10,11]. It is a commensal organism that is part of the normal flora of the skin and mucous membranes of dogs, colonizing from 25% up to nearly 70% of healthy dogs [12,13]. Despite its commensal role, *S. pseudintermedius* can be an opportunistic pathogen, causing, in addition to skin infections, otitis externa and infections in the urinary, respiratory and reproductive tracts [2,14]. Its commensal role in cats, rabbits and horses is not well established. For example, this species presents a low adherence to feline corneocytes and has been found transiently in the skin of healthy cats [15]. *S. pseudintermedius* has been isolated from circa 10% and 27% of healthy or diseased cats, respectively [16].

Regarding *S. aureus*, it is also an agent of pyoderma in companion animals, although in a lower frequency. Colonization of companion animals by this species is usually transient and associated with close contact with humans or other animals colonized by *S. aureus* [1,17]. Studies have reported a frequency of colonization of about 10% of healthy dogs [18] and 25% of healthy cats [16]. Like *S. pseudintermedius*, infections by *S. aureus* are mostly of endogenous origin and can affect dogs, cats, horses and rabbits [19,20]. *S. coagulans*, previously classified as *Staphylococcus schleiferi* subsp. *coagulans* [21], is found in the skin of healthy and diseased dogs, in frequencies of 4% and 12% [22], respectively, and is rarely reported in either healthy or diseased cats (<1%) [16]. In immunocompromised dogs, it can cause pyoderma, otitis or urinary tract infections [23,24].

Superficial skin infections are usually treated with topical therapy, based on the use of biocide- or antibiotic-based shampoos, sprays or gels. For severe cases, topical therapy combined with systemic therapy is recommended [1]. Methicillin-resistant strains, which are resistant to all beta-lactam antibiotics, except fifth-generation cephalosporins (e.g., ceftaroline), are often associated with multidrug resistance (MDR) phenotypes [25,26,27]. The increasing report of skin infections caused by such strains makes the treatment of infections more challenging due to restricted therapeutic options [25,26].

CoPS have a range of virulence factors that allow them to evade the host immune system and to establish infection [2,28]. In *S. aureus* and *S. pseudintermedius*, several virulence factors have been described including leukocidins, hemolysins, adhesines and enterotoxins [28]. The virulence factors of *S. coagulans* are still scarcely studied. Yet, the occurrence of staphylococcal enterotoxins (SE) has already been described for this species [29]. Another factor contributing to virulence is the ability to form biofilms, a capacity already described for *S. pseudintermedius* [30], *S. aureus* [31] and less extensively for *S. coagulans* [32]. Biofilms are bacterial communities made up of cells that are reversibly linked together and fixed in a self-producing polymeric matrix [33]. Biofilm formation comprises four phases: attachment, where bacterial cells attach to a biotic or abiotic surface; proliferation/accumulation, when bacteria begin to multiply and accumulate at the primary adhesion site; maturation, when microcolonies evolve into macrocolonies and the biofilm acquires a three-dimensional structure; dispersal, when individual cells detach from the matrix and spread, promoting the dissemination of the bacteria within the host [34]. One of the best know biofilm formation mechanisms is the *ica*-dependent process in *Staphylococcus epidermidis*. The *ica* operon encodes the polysaccharide intercellular adhesin (PIA), which has an important role in the attachment and accumulation phases of biofilm formation. This operon has been detected in other staphylococci such as *S. aureus* [31]. Biofilms have also an impact on the management of skin infections. Biofilm-associated infections are commonly chronic and more resilient to antibiotherapy; thus, they are associated with higher rates of antimicrobial resistance [35]. Another important virulence factor is the Panton–Valentine leukocidin PVL (*S. aureus*)/Leukocidin LukI (*S. pseudintermedius*) [36,37]. These leukocidins promote the formation of a pore in the phospholipid membrane of leukocytes, leading to ion flux, apoptosis and cell death [38]. Several authors have reported a relation between their presence and skin infections [39,40,41], but their role is unclear [42]. Many of these staphylococcal virulence factors are regulated by a quorum sensing system, the *agr* system. This system is codified by the *agrABCD* operon that encodes an autoinducing peptide (AIP). The *agr* system has been described in *S. aureus* and *S. pseudintermedius* and, in both species, four types of AIP have been reported, enabling the differentiation of four *agr* types [43,44].

In this work, we aimed to characterize the virulence potential of a collection of *S. pseudintermedius*, *S. aureus* and *S. coagulans* involved in skin infections in companion animals by assessing their capacity to produce biofilm and relating this capacity with other phenotypic (methicillin/multidrug resistance) and genotypic (*agr* type, clonal lineage) traits. Representative strains were then evaluated in the *Galleria mellonella* larvae infection model, which allowed to differentiate the virulence potential of the three CoPS species.

## 2. Results

### 2.1. Biofilm Production Is a Frequent Trait of Coagulase-Positive Staphylococci Causing Skin Infections in Companion Animals

The proportion of biofilm producers varied within species (Table 1). Biofilm production was highly frequent in *S. aureus* and *S. coagulans*, detected for 52/55 (94.6%) and 24/27 (88.9%) of the isolates, respectively. A lower frequency of biofilm producers was registered for *S. pseudintermedius*, accounting for about half of the isolates tested (79/155, 51.0%). The proportion of biofilm producers and non-producers according to animal host is detailed for each CoPS in Table 1.

**Figure 1:**
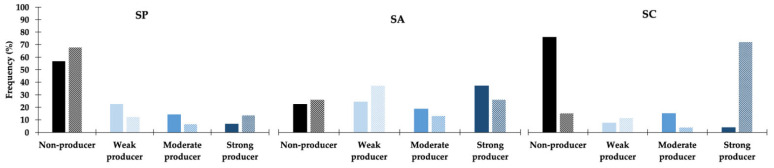
Effect of NaCl supplementation in biofilm production for the *S. pseudintermedius* (SP, *n* = 155), *S. aureus* (SA, *n* = 55) and *S. coagulans* (SC, *n* = 27) strains. Full-colored columns—TSB supplemented with 1% glucose (SA and SC) and 1% NaCl (SP); partially filled columns—TSB supplemented with 1% glucose and 3% NaCl (all species).

Biofilm production was highly affected by NaCl (Figure 1), and this effect was more pronounced for *S. coagulans*, for which 14 out of the 20 strains classified as non-producers in TSB + 1% glucose showed moderate or strong biofilm production upon supplementation of the growth medium with 3% NaCl. A distinct effect was observed for *S. aureus* and *S. pseudintermedius*, for which a small increase in the number of non-producers was registered upon increase of NaCl in the growth medium. A change to the biofilm-producing phenotype with increasing NaCl supplementation was also detected for these species but only for 4/12 and 9/88 strains, respectively.

### 2.2. Relation between Biofilm Phenotypes, Agr Types and Antimicrobial Resistance

The relation between different characteristics of the strains was evaluated using the chi-square test. For this analysis, we only considered moderate or strong biofilm producers. For *S. aureus*, the biofilm production phenotype was not statistically associated with any of the other traits tested, namely, methicillin resistance (X^2^ = 3.186; *p* = 0.074), multidrug resistance (X^2^ = 0.535; *p* = 0.734) or *agr* type (X^2^ = 3.291; *p* = 0.462).

**Figure 2:**
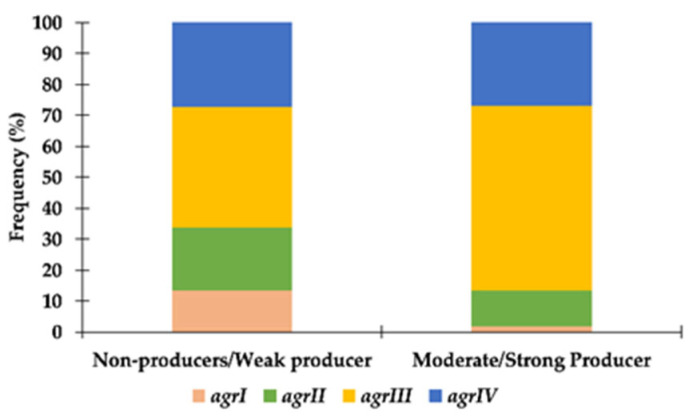
Distribution of *S. pseudintermedius* strains according to their *agr* type and capacity to produce biofilm.

For *S. pseudintermedius*, there was a statistically significant association between biofilm production and *agr* type (X^2^ = 9.674; *p* = 0.021). Comparing moderate/strong producers with weak or non-producers, strains presenting *agrIII* were more frequently categorized as moderate/strong biofilm producers, whereas strains presenting *agrI* were mainly weak/non-producers (Figure 2). The relation between biofilm production and methicillin resistance (X^2^ = 0.914; *p* = 0.334) or multidrug resistance (X^2^ = 0.109; *p* = 0.741) was not statistically significant.

### 2.3. Relation between Biofilm Phenotypes and Clonal Lineages

The most common *S. pseudintermedius* and *S. aureus* lineages include a high frequency of biofilm-producing strains. Nearly half of the *S. aureus* collection studied, gathered over a 19 year period, comprised ST22-*agrI*-MRSA isolates [45], 88.0% of which were biofilm producers. The second and third *S. aureus* predominant lineages, ST5-*agrII*-MRSA/MSSA (12.7%) and ST398-*agrI*-MRSA/MSSA (9.1%), respectively, only included biofilm producers.

For *S. pseudintermedius*, the ST71-*agrIII*-MRSP-MDR was the most common lineage among the study collection [46] and included 70.8% (17/24) of biofilm-producing strains. On the other hand, all strains (4/4) of ST157-*agrIV*-MRSP lineage, the second most common lineage, were non-biofilm producers.

Because no MLST scheme is available for *S. coagulans*, the 27 isolates were previously typed by PGFE, leading to the identification of a predominant clone (PFGE type A) that accounted for 51.9% of the collection [47]. Most *S. coagulans* of this predominant clone were strong biofilm producers (92.9%, 13/14). However, the only two MRSC strains did not produce biofilm.

These analyses suggest that biofilm production is a frequent trait of the prevalent staphylococcal clonal lineages circulating among companion animals in Portugal, most of which are already related to a high burden of antimicrobial resistance.

### 2.4. Analysis of Ica and Leukocidin-Encoding Genes across CoPS

We screened by PCR the presence of the *icaADB* genes, that are part of the *ica* operon associated with PIA production [48]. All *S. aureus* and *S. pseudintermedius* strains carried these genes, indicating a 100% frequency of *icaADB* genes in both species.

We also screened the *lukF-PV*/*lukS-PV* genes (*S. aureus*) and *lukF* gene (*S. pseudintermedius*), encoding leukocidins PVL and LukI, respectively. Similarly to *ica*, all *S. pseudintermedius* carried the *lukF* gene. However, only one *S. aureus* presented the *lukF-PV*/*lukS-PV* genes. This strain was collected from a rabbit and was the only representative of the clonal lineage ST121-*agrIV* in the study collection [45].

To complement our analysis, we carried out an in silico search of the *ica* operon genes in all the complete genomes available at the GenBank database (up to July 2022) for the three CoPS species (Supplementary material S1). The presence of the four *ica* genes (*icaA*, *icaB*, *icaC* and *icaD*) was detected for 106 out of the 107 *S. pseudintermedius* complete genomes. A similar search for *ica* genes against all *S. aureus* complete genomes (>800 genomes) also revealed their ubiquitous presence in *S. aureus*. In opposition, no *ica* genes were detected in the two *S. coagulans* genomes available.

We carried out a similar approach for the search of leukocidin-encoding genes *lukF*-*lukS* in *S. pseudintermedius* and *S. coagulans*. We conclude that for both species, these genes were encountered in all tested genomes (one-hundred and seven and two, respectively).

### 2.5. Virulence Potential of Representative CoPS Strains in the G. mellonella Infection Model

**Figure 3:**
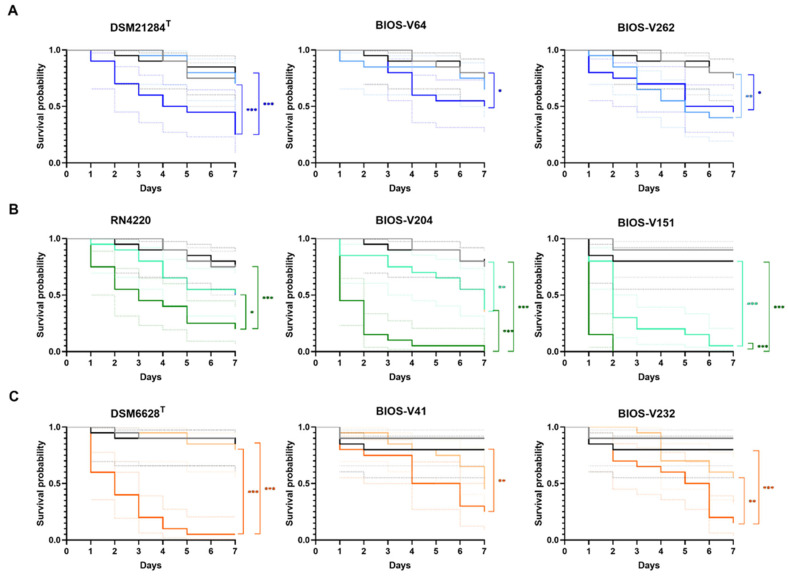
Kaplan–Meier survival analysis of *G. mellonella* infected with reference strains and biofilm-producing *S. pseudintermedius* (**A**), *S. aureus* (**B**) and *S. coagulans* (**C**) strains representative of the main clonal lineages causing animal pyoderma. Grey line: “no manipulation” control group; black line: “PBS” control group; light colors: 1 × 10^5^ CFU/larva; dark colors: 1 × 10^7^ CFU/larva. The colored dotted lines indicate the 95% confidence interval for the corresponding survival curve. Statistical differences are highlighted as follows: * *p* < 0.05; ** *p* < 0.01; *** *p* < 0.001.

The *S. pseudintermedius* clinical strains tested were BIOS-V64 and BIOS-V262, both MRSP-MDR, representing ST71-*agrIII* and ST118-*agrII*, respectively. The Kaplan–Meier survival curves and mean survival times are presented in Figure 3A and Table 2. Overall, these results show that the virulence potential varied according to the *S. pseudintermedius* infecting strain, as follows: BIOS-V262 > DSM 21284^T^ > BIOS-V64.

For *S. aureus*, the clinical strains chosen were BIOS-V204 (MRSA-ST22-*agrI*) and BIOS-V151 (MRSA-MDR-ST398-*agrI*). Overall, *S. aureus* strains showed higher virulence than *S. pseudintermedius*. Comparing the virulence potential of the three *S. aureus* strains, we could rank them as follows: BIOS-V151 > BIOS-V204 > RN4220 (Figure 3B, Table 2).

The *S. coagulans* clinical strains presented a behavior similar to *S. pseudintermedius* against *G. mellonella* (Figure 3C and Table 2). Albeit the reference strain DSM 6628^T^ displayed the highest virulence potential, a comparison between the two clinical strains showed that BIOS-V41 (MSSC-PFGE type A) presented a higher virulence potential; overall, the *S. coagulans* strains ranked as DSM 6628^T^ > BIOS-V41 > BIOS-V232.

Regardless of the infecting staphylococci or strain, the killing effect upon *G. mellonella* increased with the bacterial inoculum, as indicated by the mean larvae survival time (Table 2). The joint analysis of this parameter with the Kaplan–Meier survival curves also suggest that *S. aureus* is the species with the highest virulence potential, followed by *S. coagulans* and *S. pseudintermedius*.

## 3. Discussion

CoPs are the main cause of pyoderma in companion animals [49]. Among these, *S. pseudintermedius* is the predominant pathogen, particularly in dogs, followed by *S. aureus* and *S. coagulans*. Methicillin resistance has been increasing for these three species, causing concerns regarding the management of these infections, which often present a recurrent nature, with animals being subjected to several rounds of antibiotherapy [49].

Besides antimicrobial resistance, virulence factors allow these bacteria to be more successful on promoting infection [28]. One of the most important virulence factors in staphylococci is the production of biofilm, which is often associated with antimicrobial resistance and chronic infections [50]. Although biofilm production by animal-associated CoPS has been studied in a lesser extent than for human-associated staphylococci, the capacity to produce biofilms has already been reported for *S. pseudintermedius* and *S. coagulans* [44,51]. The molecular mechanisms of biofilm formation are described in more detailed for *S. aureus,* and it is thought that these mechanisms are similar to the ones present in other staphylococci [44,51].

In this study, we demonstrated that biofilm formation is a frequent trait in CoPS causing pyoderma in companion animals, with 65.4% (155/237) of the bacterial collection producing biofilms. Of the three species under study, *S. aureus* showed the highest capacity to form biofilms with nearly 95% producers; this is similar to other reports, which described between 90% to 100% producers [52,53,54,55,56]. *S. coagulans* was the second species for which biofilm production was more frequent, a trait detected in 88.9% (24/27) of the strains. Finally, *S. pseudintermedius* showed the lowest frequency of biofilm production, detected in half (51.0%, 79/155) of the strains. Data available in literature indicate higher frequencies of biofilm production, ranging from 90% to 100%, for *S. coagulans* [29,51] and for *S. pseudintermedius* [30,44,57,58,59,60,61]. The divergence between these results may rely on the methodology used to assess biofilm production. The crystal violet adhesion method is an effective and low-cost method that allows high-throughput detection of biofilms. However, it shows high intra- and inter-assay variation, which can be exacerbated when comparing inter-laboratorial data due to differences in the criteria used to categorize the biofilm phenotype, and other factors, such as growth media and incubation conditions [62]. In fact, our study highlights that supplementation of the growth media with a high saline concentration impacts significantly on biofilm formation. Our data also indicate that NaCl can act either as an inhibitor or inducer of biofilm production, being more notorious for biofilm induction in *S. coagulans* and biofilm inhibition in *S. aureus*. NaCl has been reported to induce the expression of the *ica* operon in *S. aureus* [63,64]. However, our observations for *S. aureus* and *S. pseudintermedius* indicate that despite a small increase in the biofilm-producing strains, the frequency of moderate/strong biofilm producers diminishes with NaCl supplementation. The *ica* operon was detected amongst all *S. aureus* and *S. pseudintermedius* strains, in accordance with results obtained by other authors [44,57,59,65,66]. Moreover, the in silico analysis corroborated the wide presence of the operon in both species. These apparently contradictory findings may suggest that production of biofilm in these strains may occur partially via an *ica*-independent mechanism that is affect by higher osmolarity environments [67]. Growth conditions have also been demonstrated to affect the chemical properties of the biofilm matrix [68], such as an increase in biofilm hydrophobicity in the presence of lower NaCl concentrations, which impacts biofilm adherence properties [69]. Further functional studies should highlight the similarities and differences of the process of biofilm formation in CoPS.

No statistical associations could be established between the biofilm phenotype and antimicrobial resistance phenotypes (methicillin resistance and multidrug resistance) or with clonal lineage (ST, *agr* type) for *S. aureus.* In opposition, for *S. pseudintermedius*, an association was found between biofilm production and *agr* type, namely, a higher frequency of *agrIII* among biofilm-producing strains and a lower frequency of *agrI* among non-producing strains. These results differ from the findings of Little and colleagues, which reported a relation between *agrIII* and *agrII* and the non-biofilm-producing phenotype [44]. No associations were encountered between biofilm production and antimicrobial resistance phenotypes. These statistical analyses were not extended to *S. coagulans*, but noteworthily, the only two MRSC strains do not form biofilms.

Pore-forming leukocidins are major virulence factors in staphylococci. In *S. aureus* and *S. coagulans*, this leukocidin is encoded by *lukF-PV*/*lukS-PV* genes [36,70] and, in *S. pseudintermedius,* by *lukF*- *lukS* genes [37]. Some authors associate these toxins to skin infections, although this relation is not always well established [39,40,41]. All *S. pseudintermedius* strains carried the gene *lukF*, suggesting that this gene may be part of the core genome of this species since the studied collection is genetically diverse [46]. This observation is further supported by the in silico detection of both *lukF* and *lukS* genes in all available *S. pseudintermedius* complete genomes. For *S. aureus*, only one strain isolated from a rabbit presented the *lukF-PV*/*lukS-PV* genes. This strain belongs to ST121-*agrI*, a clonal lineage frequently associated with rabbits [71].

The use of animal models in scientific research is essential to understand the interaction between the pathogen and its host [72]. The animal model *G. mellonella* has been increasingly used in recent years [73]. This model has several advantages for scientific research purposes, namely: (i) the larva can be kept at 37 °C; (ii) the innate immune system of the larva shows similarities with the innate immune system of mammals; (iii) does not require major adaptations at the laboratory level; (iv) does not require ethical approval [74]. One limitation in the use of *G. mellonella* is the lack of centers specializing in the supply of larvae for research purposes. Thus, the larvae used in many studies are purchased from suppliers specialized in breeding this species for pet food, which potentiates genotypic differences among larvae. The other limiting factor is uncontrolled breeding and maintenance conditions, which can influence the *G. mellonella* susceptibility to infections [73,75]. Nevertheless, this animal model has already been proved valuable to study the virulence of several bacteria, including *S. aureus* [76,77,78,79], and to a lesser extent, *S. pseudintermedius* and *S. coagulans* [80]. The infection assays conducted in these studies varied in several parameters such as the time of larvae follow-up, ranging from three up to 10 days [76,77,78,79,80,81,82], and bacterial inoculum, which ranged from 2 × 10^4^ CFU/larva [76], 5 × 10^6^ CFU/larva [77] to 2 × 10^7^ CFU/larva [80]. In this study, we followed larvae survival for 7 days and used two inoculums: 1 × 10^5^ and 1 × 10^7^ CFU/larva. In these conditions, we were able to monitor and differentiate larvae mortality between strains or CoPS species.

The three CoPS species presented different virulence potential in the *G. mellonella* infection model. Overall, *S. aureus* strains showed higher virulence potential than the *S. pseudintermedius* and *S. coagulans* strains, as observed in the lower survival probabilities and lower mean survival times (Figure 3 and Table 2). A report by Canovas and colleagues also demonstrated a higher virulence potential of *S. aureus* when compared with *S. coagulans* [80]. In particular, the two *S. aureus* clinical strains studied demonstrated a statistically significant higher killing activity than the reference strain. Both clinical strains are MRSA and strong biofilm producers and belong to clonal lineages that are prevalent in our study collection. Strain BIOS-V151 belongs to ST398-*agrI* [45], a lineage frequently associated with food-producing or companion animals [19]. On the other hand, BIOS-V204 belongs to ST22-*agrI*, the predominant lineage in our pyoderma-related *S. aureus* collection as well as in *S. aureus* from skin infections in humans in the community [83], highlighting concerns on the sharing of such strains between animals and humans.

*S. pseudintermedius* and *S. coagulans* presented similar virulence potential. For both species, the biofilm-producing clinical strains only showed increased virulence potential at the higher inoculum tested, 10^7^ CFU/larva. This observation is of relevance considering that skin infections are characterized by a high bacterial burden at the site of infection. The strains tested represent bacteria with a high capacity to produce biofilms from relevant circulating clonal lineages. *S. coagulans* BIOS-V41 belongs to the most common lineage found amongst the 19-year timespan of our collection [47]. *S. pseudintermedius* BIOS-V64 is a moderate biofilm producer belonging to MRSP-MDR-ST71-*agrIII*, the predominant lineage in the *S. pseudintermedius* collection and one of the most prevalent causing canine infections in several European countries [46,84]. An increased virulence potential at both inoculums tested was only observed for *S. pseudintermedius* BIOS-V262, a moderate biofilm producer that belongs to MRSP-MDR-ST118-*agrII*. This lineage is a double locus variant of ST258, a clonal lineage that is replacing ST71 in some European countries [85], raising concerns on the dissemination of ST258 and its variants and their potential higher virulence potential associated with skin infections.

## 4. Materials and Methods

### 4.1. Bacterial Isolates

The study included 155 *S. pseudintermedius* (isolated from 141 dogs, 3 cats and one rabbit), 55 *S. aureus* (isolated from 27 dogs, 18 cats, four rabbits, one horse and one unknown host) and 27 *S. coagulans* (from 26 dogs) strains associated with skin infections in companion animals collected over a period of 19 years (1999–2018) at a veterinary research laboratory providing diagnostic services for a veterinary teaching hospital and private veterinary clinics in the Lisbon area (1999–2018) and at a private veterinary diagnostic clinic (2017–2018). A few strains were isolated from the same animal host, as follows: 12 *S. pseudintermedius* strains were isolated from six dogs (two strains/dog) and six other *S. pseudintermedius* strains were isolated from two dogs (three strains/dog); eight *S. aureus* strains were isolated from two dogs, one cat and one rabbit (two strains/animal); two *S. coagulans* were isolated from the same dog. All strains have been previously characterized regarding their antimicrobial susceptibility profiles, and their clonal lineages were determined by PFGE and/or MLST [45,46,47]. The main strain characteristics are summarized in Supplementary Material S2. All isolates were grown in tryptone soya broth or agar (TSB/TSA, Thermo Scientific™ Oxoid™, Basingstoke, UK) at 37 °C (for broth cultures). Bacterial stocks were kept at −80 °C in TSB supplemented with 10% (*v*/*v*) glycerol (Sigma-Aldrich, Saint Louis, MO, USA). The type strains *S. pseudintermedius* DSM 21284^T^ and *S. coagulans* DSM 6628^T^, and the reference strains *S. aureus* RN4220, *S. epidermidis* ATCC^®^12228™ and ATCC^®^35984™ (*S. epidermidis* RP62a) were included in the study as controls.

### 4.2. Assessment of Biofilm Formation

The capacity of the strains to produce biofilm was assessed by the crystal violet adhesion method in 96-well flat-bottom tissue culture plates (Orange Scientific, Braine-l’Alleud, Belgium) [62,86]. Briefly, strains were cultured in TSB for 24 h at 37 °C with no agitation. A cellular suspension adjusted to 5 × 10^7^–1 × 10^8^ CFU/mL in TSB was prepared, diluted in 1:100 in supplemented TSB and 0.2 mL aliquots distributed in quadruplicates in the microtiter plates. Different growth conditions were evaluated as follows: *S. pseudintermedius* [TSB + 1% glucose (Sigma-Aldrich, Saint Louis, MO, USA) + 1% or 3% NaCl (Merck, Darmstadt, Germany)]; *S. aureus* and *S. coagulans* (TSB + 1% glucose w/wo 3% NaCl). After incubation for 24 h at 37 °C, the wells content was discarded carefully with a multichannel micropipette and washed thrice with 0.1 mL phosphate buffered saline (PBS, Sigma-Aldrich, Saint Louis, MO, USA). Adherent cells were fixed with 0.15 mL methanol 99% (Sigma-Aldrich, Saint Louis, MO, USA) for 20 min and after an air-dry overnight period, the biofilm biomass was dyed with 0.15 mL 0.1% (*w*/*v*) crystal violet (Sigma-Aldrich, Saint Louis, MO, USA) for 15 min. Following, the microtiter plates were subjected to two wash cycles in two water baths and air-dried for at least 90 min. The bound crystal violet was solubilized with 0.15 mL of 33% (*v*/*v*) acetic acid (Sigma-Aldrich, Saint Louis, MO, USA) for 30 min and the associated optical density was measured at 570 nm (well area mode) in a Synergy^HT^ apparatus (Biotek, Winooski, VT, USA). Each assay included, in quadruplicates, the control strains *S. epidermidis* ATCC^®^12228™ (*ica* ^-^), *S. epidermidis* ATCC^®^35984™ (*ica* ^+^) and the corresponding reference strains (*S. pseudintermedius* DSM 21284^T^, *S. aureus* RN4220, *S. coagulans* DSM 6628^T^), as well as a negative control (supplemented TSB) and a blank (33% acetic acid). Biofilm production was categorized according to Stepanović criteria [62,86], which establishes a cut-off value (ODc) defined as the geometric media of OD_570_ of the negative control (supplemented TSB) + 3x the corresponding standard deviation (SD). Strains were characterized as follows: OD_strain_ < ODc, biofilm non-producers; ODc < OD_strain_ < 2x ODc, weak producers; 2x ODc < OD_strain_ < 4x ODc, moderate producers; OD_strain_ > 4x OD, strong producers. Each assay was performed, at least, in duplicate and, to minimize intra- and inter-assay variability, an assay was only validated when associated with an SD value <20% of the corresponding geometric mean (either considering an individual assay or duplicates) and assigning the strain to the same category. A strain was considered a biofilm producer if assigned to the weak, moderate or strong phenotypes in, at least, one of the growth conditions tested.

Statistical analyses were performed in SPSS v26.0 (IBM^®^, Armonk, NY, USA) to verify associations between biofilm production and *agr* type and resistance traits (methicillin resistance and MDR phenotype) using the chi-square test. Statistical significance was considered for *p* < 0.05.

### 4.3. Isolation of Total DNA, Agr Typing and Identification of Ica Genes by PCR

Total DNA from each strain was extracted by the boiling method as described by Alexopoulou and colleagues [87].

*Agr* typing was performed for all *S. pseudintermedius* and *S. aureus*. For *S. pseudintermedius*, the *agrD* gene was amplified by PCR and sequenced using the primers described in Supplementary Material S2. The nucleotide sequences were then compared with the four reference sequences of the four agr types, as follows: *agrI* (accession no. EU157336); *agrII* (accession no. EU157366); *agrIII* (accession no. EU157334); *agrIV* (accession no. EU157330) [88]. For *S. aureus*, *agr* typing was carried out following a multiplex PCR approach, as described by Lina and colleagues [89].

All strains were screened by PCR for the presence of the *icaADB* genes associated with biofilm production using primers specific for each species (Supplementary Material S2). The presence of the determinants *lukF-PV* and *lukS-PV* encoding PVL or *lukF* encoding LukI was screened by PCR using the primers also described in Supplementary Material S2 [90]. Primers were designed using Primer-blast [91] based on the genome of *S. pseudintermedius* HKU10-03 (accession no. CP002439) and *S. aureus* RN4220 (accession no. CP076105). All primers used were synthesized by Invitrogen (Waltham, MA, USA).

### 4.4. In Silico Search of the Presence of the Ica Operon and PVL/LukI Determinants in Staphylococcal Genomes

The presence of *ica* and leukocidin-encoding genes was evaluated in the complete genomes available at GenBank database (https://www.ncbi.nlm.nih.gov/genbank/, accessed on 30 August 2022, National Library of Medicine, Bethesda, MD, USA) for *S. pseudintermedius* (*n* = 107), *S. aureus* (*n* > 800) and *S. coagulans* (*n* = 2, only chromosome sequences available).

Using the Blast tool (https://blast.ncbi.nlm.nih.gov/Blast.cgi, accessed on 30 August 2022, National Library of Medicine, Bethesda, MD, USA), those genomes were queried for the presence of *lukF-lukS* (*S. pseudintermedius, S. coagulans*) and the four genes of the *ica* operon (*icaA*, *icaB*, *icaC* and *icaD*) (all three species). These searches were carried out using the nucleotide sequences of the respective genes of the representative genomes *S. pseudintermedius* SP_11304-3A (accession no. NZ_CP065921.1) and *S. aureus* NCTC 8325 (accession no. NC_007795.1).

### 4.5. Assessment of Virulence Potential in a G. mellonella Infection Model

The *G. mellonella* infection model has been applied to evaluate the virulence of different pathogens [81]. In this study, *G. mellonella* was used to assess the virulence potential of representative strains of each staphylococcal species in study (three strains per species, corresponding to one reference and two clinical strains). The strains tested were biofilm producers and presented additional relevant phenotypic and genotypic characteristics, such as methicillin resistance and belonging to predominant clonal lineages (Table 3). The virulence potential of each strain was tested using two different bacterial inoculums, 10^5^ and 10^7^ CFU/larva.

*G. mellonella* at the larva stage were acquired from a pet-food supplier (Reptimundo, Faro, Portugal). The larvae were maintained in the dark at room temperature and were used within one week [92]. Prior to each assay, the larvae were acclimatized overnight to 37 °C. The bacterial inoculum was prepared by overnight growth in TSB at 37 °C and 180 rpm; the bacterial cells were collected, washed thrice in PBS, resuspended and adjusted in PBS to 5 × 10^8^ CFU/mL (corresponding to OD_600_ of 0.25–0.26 (*S. aureus*) and 0.29–0.31 (*S. pseudintermedius* and *S. coagulans*)). The CFU/mL was verified by plating aliquots of the cellular suspension in TSA. An aliquot was further diluted 1:100 in PBS to achieve 5 × 10^6^ CFU/mL. For each cellular suspension, serial dilutions were prepared up to 10^−7^ and an aliquot of 0.1 mL was plated in TSA (VWR International LLC, Radnor, PA, USA) for bacteria enumeration.

Groups of ten larvae, selected based on similar weight (200–250 mg/larvae) and size (20–25 mm), no melanization and presence of motility [93] were restrained [92] and inoculated with 0.02 mL of (i) PBS (control), (ii) 5 × 10^8^ CFU/mL (corresponding to 1 × 10^7^ CFU/larva) and (iii) 5 × 10^6^ CFU/mL (corresponding to 1 × 10^5^ CFU/larva) using an insulin syringe (Braun Omnican^®^ 100, B. Braun, Melsungen, Germany). A fourth group of ten non-manipulated larvae was included per assay. The larvae were kept at 37 °C with food (bee wax shavings (Reptimundo)) [94], and larvae survival was evaluated each 24 h post-infection for seven days. Dead larvae (full melanization, lack of motility) were removed at each timepoint, transferred to a falcon tube and placed in a secondary containment vessel at −20 °C overnight prior to disposal for incineration. The virulence potential of each staphylococcal strain was evaluated in two independent infection assays. Data were analyzed by Kaplan–Meier survival curves and the mean survival time was determined using GraphPad Prism v 8.0.1 (San Diego, CA, USA). Survival rates between groups were compared with the Log-Rank (Mantel–Cox) test. Statistical significance was considered for *p*-values <0.05.

## 5. Conclusions

This study provides evidence on the high prevalence of biofilm production by CoPS strains causing skin infections in companion animals in Portugal. Worrisomely, production of biofilm was detected amongst predominant *S. pseudintermedius* and *S. aureus* clonal lineages associated with a high burden of antimicrobial resistance. Additionally, we highlight an increased virulence potential for these antimicrobial-resistant CoPS strains, raising concerns on the future management of skin infection in companion animals and strengthening the need for improved surveillance of these pathogens.

## Figures and Tables

**Table 1:** Distribution of biofilm production phenotype for the *S. pseudintermedius* (SP), *S. aureus* (SA) and *S. coagulans* (SC) strains in the different growth conditions tested. The proportion of biofilm producers and non-producers according to animal host is also detailed for each CoPS. A strain was considered a biofilm producer if it showed weak, moderate or strong production of biofilm in either one or both conditions tested. A strain was considered a non-producer of biofilm if categorized as non-producer in the two conditions tested.

	Biofilm Non-Producers *n* (%)		Biofilm Producers, *n* (%), at the Following Condition(s) *					
			One Growth Condition				Two Growth Conditions	
			w/o NaCl (SA/SC) w/1% NaCl (SP)		w/3% NaCl (SA/SC/SP)		w/o NaCl; w/3% NaCl (SA/SC) w/1% NaCl; w/3% NaCl (SP)	
**SP** (*n* = 155)	76 (49.0%)	73 from dogs 2 from cats 1 from rabbits	29 (18.7%)	28 from dogs 1 from cats	12 (7.8%)	12 from dogs	38 (24.5%)	38 from dogs
**SA** (*n* = 55)	3 (5.4%)	1 from dogs 1 from cats 1 from rabbits	11 (20.0%)	6 from dogs 5 from cats	9 (16.4%)	5 from dogs 2 from cats 2 from rabbits	32 (58.2%)	17 from dogs 11 from cats 2 from rabbits 1 from horses 1 unknown
**SC** (*n* = 27)	3 (11.1%)	3 from dogs	1 (3.7%)	1 from dogs	17 (63.0%)	17 from dogs	6 (22.2%)	6 from dogs

* For each species, biofilm production was evaluated in two growth conditions as follows: *S. pseudintermedius*, 1% glucose with 1% NaCl or 3% NaCl; *S. aureus*/*S. coagulans*, 1% glucose without NaCl or with 3% NaCl.

**Table 2:** Mean survival time of *G. mellonella* larvae infected with representative biofilm-producing CoPS strains.

Species/Strain	Mean Larvae Survival Time (Days) after Infection with:	
	10^5^ CFU/Larva	10^7^ CFU/Larva
***S. pseudintermedius***		
DSM 21284^T^	>7	4.5
BIOS-V64	>7	7
BIOS-V262	5	6
***S. aureus***		
RN4220	7	3
BIOS-V204	7	1
BIOS-V151	2	1
***S. coagulans***		
DSM 6628^T^	>7	2
BIOS-V41	7	5
BIOS-V232	>7	5.5

The mean larvae survival time for the controls “no manipulation” and “PBS” was >7 days in all assays.

**Table 3:** Main characteristics of the biofilm-producing strains studied on the *G. mellonella* infection assays [45,46,47].

Strain	Characteristics
***S. pseudintermedius***	
DSM 21284^T^	Biofilm producer; MSSP/MDR; ST63-*agrIV*
BIOS-V64	Biofilm producer; MRSP/MDR; ST71-*agrIII*
BIOS-V262	Biofilm producer; MRSP/MDR; ST118-*agrII*
***S. aureus***	
RN4220	Biofilm producer; MSSA; ST8-*agrI*
BIOS-V151	Biofilm producer; MRSA/MDR; ST398-*agrI*
BIOS-V204	Biofilm producer; MRSA/non-MDR; ST22-*agrI*
***S. coagulans***	
DSM 6628^T^	Biofilm producer; MSSC
BIOS-V41	Biofilm producer; MSSC; PFGE type A
BIOS-V232	Biofilm producer; MSSC; PFGE type G

## Data Availability

All relevant data have been provided in the paper. Raw data can also be provided by the authors upon reasonable request.

## Supplementary Materials

The following are available online at https://www.mdpi.com/article/10.3390/antibiotics11101339/s1, Supplementary material S1: In silico search of *ica* genes and leukocidin determinants in complete genomes of *S. pseudintermedius*, *S. aureus* and *S. coagulans*; Supplementary material S2: Characteristics of the staphylococcal isolates studied and list of the primers used.
