# Supplementary material for: Virulence Potential of Biofilm-Producing Staphylococcus pseudintermedius, Staphylococcus aureus and Staphylococcus coagulans Causing Skin Infections in Companion Animals
Source: Antibiotics (Basel). 2022 Sep 30;11(10):1339. doi: 10.3390/antibiotics11101339 (PMC9598800; doi:10.3390/antibiotics11101339)
Supplement: Supplementary file 1 [file antibiotics-11-01339-s001.zip › Supplementary material S2.pdf]

Article

Virulence Potential of Biofilm-Producing *Staphylococcus pseudintermedius*, *Staphylococcus aureus* and *Staphylococcus coagulans* Causing Skin Infections in Companion Animals

Mariana Andrade, Ketlyn Oliveira, Catarina Morais, Patrícia Abrantes, Constança Pomba, Adriana E. Rosato, Isabel Couto and Sofia Santos Costa

Table S1. Main characteristics of *S. coagulans* (*n* = 27) isolates associated with canine skin infections included in the study [47]. Antimicrobial resistance profile and clonal lineages were determined previously.

Isolate	Biological sample	Resistance profile	PFGE type	Host	Year	Lab
BIOS-V1[(1)]	skin swab	MSSC, non-MDR	F1	Dog	2015	Lab1
BIOS-V2	skin swab	MSSC, non-MDR	A1	Dog	2012	Lab1
BIOS-V3	skin swab	MSSC, non-MDR	F3	Dog	2014	Lab1
BIOS-V9	skin swab	MSSC, non-MDR	C	Dog	2018	Lab1
BIOS-V35	skin swab	MSSC, non-MDR	A8	Dog	2018	Lab1
BIOS-V41	perianal skin swab	MSSC, non-MDR	A6	Dog	2018	Lab1
BIOS-V42	skin swab	MSSC, non-MDR	H	Dog	2001	Lab1
BIOS-V43	skin swab	MSSC, non-MDR	I	Dog	2004	Lab1
BIOS-V44	skin swab	MSSC, non-MDR	G1	Dog	2005	Lab1
BIOS-V45	perianal fistula swab	MSSC, non-MDR	A1	Dog	2008	Lab1
BIOS-V46	skin swab	MSSC, non-MDR	B	Dog	1999	Lab1
BIOS-V47	skin swab	MSSC, non-MDR	A3	Dog	2003	Lab1
BIOS-V51	skin swab	MSSC, non-MDR	A3	Dog	2007	Lab1
BIOS-V91	axillar skin swab	MSSC, MDR	A3	Dog	2004	Lab1
BIOS-V93	skin swab	MSSC, non-MDR	G1	Dog	2007	Lab1
BIOS-V94	skin swab	MSSC, non-MDR	A3	Dog	2007	Lab1
BIOS-V95	skin swab	MSSC, non-MDR	A3	Dog	2013	Lab1
BIOS-V98	skin swab	MSSC, non-MDR	A7	Dog	2015	Lab1
BIOS-V107	skin swab	MSSC, non-MDR	A2	Dog	2015	Lab1
BIOS-V126[(1)]	skin swab	MSSC, non-MDR	F2	Dog	2016	Lab1
BIOS-V191	skin swab	MRSC, non-MDR	D	Dog	2018	Lab2
BIOS-V205	skin swab	MSSC, MDR	A5	Dog	2018	Lab2
BIOS-V209	skin swab	MSSC, non-MDR	---	Dog	2018	Lab2
BIOS-V232	epidermal collarette	MSSC, non-MDR	G2	Dog	2018	Lab2
BIOS-V243	skin swab	MSSC, non-MDR	A3	Dog	2018	Lab2
BIOS-V265	skin swab	MSSC, non-MDR	A4	Dog	2018	Lab2
BIOS-V289	interdigital skin swab	MSSC, non-MDR	E	Dog	2018	Lab2

The numbers in brackets represent strains collected from the same animal. MSSC: methicillin-susceptible *Staphylococcus coagulans;* MRSC: methicillin-resistant *Staphylococcus coagulans;* MDR: multidrug resistant.

Table S2. Main characteristics of *S. aureus* (*n* = 55) isolates associated with canine skin infections included in the study [45]. Antimicrobial resistance profile and clonal lineages were determined previously.

Isolate	Biological sample	Resistance profile	Clonal lineage	*agr* type	Host	Year	Lab
BIOS-V4	skin swab	MRSA, MDR	398	I	Dog	2013	Lab1
BIOS-V5	skin swab	MRSA, non-MDR	22	I	Dog	2014	Lab1
BIOS-V6	skin swab	MRSA, non-MDR	22	I	Dog	2014	Lab1
BIOS-V19	skin swab	MRSA, non-MDR	22	I	Cat	2012	Lab1
BIOS-V20	skin swab	MRSA, non-MDR	22	I	Cat	2012	Lab1
BIOS-V21	skin swab	MSSA, non-MDR	97	I	Cat	2014	Lab1
BIOS-V22	skin swab	MRSA, non-MDR	22	I	Cat	2012	Lab1
BIOS-V30	skin swab	MRSA, MDR	22	I	Dog	2014	Lab1
BIOS-V31	skin swab	MSSA, non-MDR	188	I	Dog	2018	Lab1
BIOS-V60	skin swab	MSSA, non-MDR	816	II	Horse	2001	Lab1
BIOS-V61	skin swab	MSSA, non-MDR	5	II	Dog	2003	Lab1
BIOS-V62	skin swab	MSSA, non-MDR	15	II	Cat	2005	Lab1
BIOS-V63	paw swab	MRSA, non-MDR	22	I	Dog	2008	Lab1
BIOS-V70	skin swab	MRSA, non-MDR	5	II	Dog	2015	Lab1
BIOS-V74(2)	pustule liquid	MSSA, non-MDR	5	II	Dog	2003	Lab1
BIOS-V75	skin swab	MSSA, non-MDR	72	I	Dog	2005	Lab1
BIOS-V76	skin swab	MRSA, non-MDR	22	I	Cat	2012	Lab1
BIOS-V85(2)	pustule liquid	MSSA, non-MDR	5	II	Dog	2003	Lab1
BIOS-V118	skin swab	MRSA, non-MDR	6535	II	Dog	2007	Lab1
BIOS-V128	skin swab	MSSA, non-MDR	5	II	Cat	2018	Lab1
BIOS-V129	skin swab	MSSA, non-MDR	121	IV	Rabbit	2003	Lab1
BIOS-V147	skin swab	MSSA, non-MDR	398	I	Rabbit	2017	Lab2
BIOS-V150	skin swab	MRSA, non-MDR	22	I	Dog	2017	Lab2
BIOS-V151	skin swab	MSSA, MDR	398	I	Cat	2017	Lab2
BIOS-V153	skin swab	MRSA, MDR	105	II	Dog	2018	Lab2
BIOS-V154	skin swab	MRSA, non-MDR	22	I	Cat	2018	Lab2
BIOS-V155(3)	skin swab	MRSA, non-MDR	22	I	Cat	2018	Lab2
BIOS-V156	skin swab	MSSA, non-MDR	6565	I	Dog	2018	Lab2
BIOS-V157	skin swab	MRSA-non-MDR	6566	I	Dog	2018	Lab2
BIOS-V158	skin swab	MSSA, non-MDR	7	I	Cat	2017	Lab2
BIOS-V159	skin swab	MRSA, non-MDR	22	I	Cat	2018	Lab2
BIOS-V160	skin swab	MRSA, non-MDR	5	II	Cat	2018	Lab2
BIOS-V161	skin swab	MRSA, non-MDR	22	I	Rabbit	2018	Lab2
BIOS-V168	skin swab	MSSA, non-MDR	72	I	Cat	2018	Lab2
BIOS-V172	skin swab	MSSA, non-MDR	5	II	Dog	2018	Lab2
BIOS-V178	skin swab	MSSA, MDR	398	I	Cat	2018	Lab2

The numbers in brackets represent strains collected from the same animal. MSSA: methicillin-susceptible *Staphylococcus aureus;* MRSA: methicillin-resistant *Staphylococcus aureus;* MDR: multidrug resistant.

Table S2. Continuation.

Isolate	Biological sample	Resistance profile	Clonal lineage	*agr* type	Host	Year	Lab
BIOS-V183	skin swab	MRSA, non-MDR	22	I	Dog	2017	Lab2
BIOS-V184(3)	skin swab	MRSA, non-MDR	22	I	Cat	2018	Lab2
BIOS-V185	skin swab	MRSA, non-MDR	22	I	Dog	2018	Lab2
BIOS-V186	skin swab	MRSA, MDR	22	I	Dog	2018	Lab2
BIOS-V187	skin swab	MRSA, non-MDR	22	I	Dog	2018	Lab2
BIOS-V200(4)	skin swab	MRSA, non-MDR	22	I	Dog	2018	Lab2
BIOS-V201(4)	skin swab	MRSA, non-MDR	22	I	Dog	2018	Lab2
BIOS-V202	skin swab	MRSA, non-MDR	22	I	Cat	2018	Lab2
BIOS-V203	skin swab	MSSA, non-MDR	398	I	Dog	2018	Lab2
BIOS-V204(5)	skin swab	MRSA, non-MDR	22	I	Rabbit	2017	Lab2
BIOS-V245	skin swab	MSSA, non-MDR	1	III	Dog	2018	Lab2
BIOS-V250	skin swab	MSSA, non-MDR	1	III	Dog	2018	Lab2
BIOS-V255	skin swab	MRSA, MDR	105	II	Dog	2018	Lab2
BIOS-V257	skin swab	MSSA, non-MDR	15	II	Cat	2017	Lab2
BIOS-V258(5)	skin swab	MRSA, non-MDR	22	I	Rabbit	2017	Lab2
BIOS-V279	skin swab	MSSA, non-MDR	72	I	Cat	2018	Lab2
BIOS-V295	skin swab	MSSA, non-MDR	72	I	U	2018	Lab2
BIOS-V296	skin swab	MRSA, non-MDR	22	I	Dog	2018	Lab2
BIOS-V300	skin swab	MRSA, MDR	22	I	Dog	2018	Lab2

The numbers in brackets represent strains collected from the same animal. MSSA: methicillin-susceptible *Staphylococcus aureus*; MRSA: methicillin-resistant *Staphylococcus aureus;* MDR: multidrug resistant; U= unknown source.

Table S3. Main characteristics of *S. pseudintermedius* (*n* = 155) isolates associated with canine skin infections included in the study [46].

Isolate	Biological sample	*agr* type	Host	Year	Lab
BIOS-V7	skin swab	IV	Dog	2015	Lab1
BIOS-V10	skin swab	IV	Dog	2018	Lab1
BIOS-V11(7)	skin swab	III	Dog	2018	Lab1
BIOS-V12(9)	skin swab	III	Dog	2018	Lab1
BIOS-V13	paw swab	I	Dog	2018	Lab1
BIOS-V14	skin swab	III	Dog	2018	Lab1
BIOS-V15	skin swab	III	Dog	2018	Lab1
BIOS-V16	interdigital swab	II	Dog	2018	Lab1
BIOS-V18	skin swab	II	Dog	2018	Lab1
BIOS-V25	skin swab	IV	Dog	2016	Lab1
BIOS-V26	biopsy	IV	Dog	2017	Lab1
BIOS-V27	skin swab	IV	Dog	2018	Lab1

The numbers in brackets represent strains collected from the same animal. MSSP: methicillin-susceptible *Staphylococcus pseudintermedius;* MRSP: methicillin-resistant *Staphylococcus pseudintermedius;* MDR: multidrug resistant.

Table S3. Continuation.

Isolate	Biological sample	*agr* type	Host	Year	Lab
BIOS-V28	skin swab	III	Dog	2018	Lab1
BIOS-V29	interdigital swab	I	Dog	2018	Lab1
BIOS-V32	skin swab	I	Dog	2018	Lab1
BIOS-V34(7)	skin swab	III	Dog	2018	Lab1
BIOS-V36	skin biopsy	III	Dog	2017	Lab1
BIOS-V37(10)	skin swab	IV	Dog	2018	Lab1
BIOS-V38	skin swab	III	Dog	2018	Lab1
BIOS-V39	skin biopsy	III	Dog	2016	Lab1
BIOS-V40	skin swab	III	Dog	2016	Lab1
BIOS-V48	secretion	III	Dog	2014	Lab1
BIOS-V49	skin swab	II	Dog	2015	Lab1
BIOS-V50(11)	pustule	II	Dog	2015	Lab1
BIOS-V52(10)	skin biopsy	III	Dog	2017	Lab1
BIOS-V53(6)	skin swab	III	Dog	2015	Lab1
BIOS-V54	skin swab	III	Dog	2018	Lab1
BIOS-V55	skin swab	IV	Dog	2014	Lab1
BIOS-V56	skin swab	I	Dog	2014	Lab1
BIOS-V57	skin swab	IV	Dog	2015	Lab1
BIOS-V58(12)	skin swab	III	Dog	2014	Lab1
BIOS-V59	skin swab	I	Dog	2017	Lab1
BIOS-V64	interdigital swab	III	Dog	2018	Lab1
BIOS-V65	skin swab	IV	Dog	2018	Lab1
BIOS-V66	lip crease swab	III	Dog	2015	Lab1
BIOS-V67	skin swab	IV	Dog	2018	Lab1
BIOS-V68	armpit skin swab	IV	Dog	2015	Lab1
BIOS-V71	pustule swab	IV	Dog	2015	Lab1
BIOS-V72	skin swab	I	Dog	2018	Lab1
BIOS-V73	skin swab	IV	Dog	2017	Lab1
BIOS-V77	swab	IV	Dog	2015	Lab1
BIOS-V78	scrotal swab	III	Dog	2015	Lab1
BIOS-V79	skin swab	I	Dog	2015	Lab1
BIOS-V82(11)	epidermal scab swab	III	Dog	2015	Lab1
BIOS-V83	abdominal area pustule swab	III	Dog	2015	Lab1
BIOS-V84	pustule swab	IV	Dog	2015	Lab1
BIOS-V87	swab	III	Dog	2014	Lab1
BIOS-V88(12)	scab swab	IV	Dog	2015	Lab1
BIOS-V89(13)	skin granuloma	IV	Dog	2015	Lab1
BIOS-V90	skin swab	III	Dog	2015	Lab1

The numbers in brackets represent strains collected from the same animal. MSSP: methicillin-susceptible *Staphylococcus pseudintermedius;* MRSP: methicillin-resistant *Staphylococcus pseudintermedius;* MDR: multidrug resistant.

Table S3. Continuation.

Isolate	Biological sample	*agr* type	Host	Year	Lab
BIOS-V92	skin swab	II	Dog	2005	Lab1
BIOS-V96(8)	swab	IV	Dog	2015	Lab1
BIOS-V97	suture swab	III	Dog	2015	Lab1
BIOS-V99(13)	chronic wound swab	III	Dog	2015	Lab1
BIOS-V101	skin swab	II	Dog	2017	Lab1
BIOS-V102	skin swab	I	Dog	2015	Lab1
BIOS-V103	interdigital swab	II	Dog	2015	Lab1
BIOS-V104	fistula swab	III	Dog	2014	Lab1
BIOS-V105(6)	skin swab	II	Dog	2017	Lab1
BIOS-V106	deep biopsy	III	Dog	2017	Lab1
BIOS-V108	skin granuloma	III	Dog	2015	Lab1
BIOS-V110	skin swab	I	Dog	2015	Lab1
BIOS-V113	abdominal area pustule	III	Dog	2018	Lab1
BIOS-V114	dermatitis fistula in hind limb	I	Dog	2015	Lab1
BIOS-V116(8)	skin pustules	IV	Dog	2015	Lab1
BIOS-V117	skin swab	II	Dog	2018	Lab1
BIOS-V119	skin swab	III	Dog	2018	Lab1
BIOS-V120	skin swab	III	Dog	2017	Lab1
BIOS-V121	skin swab	II	Dog	2018	Lab1
BIOS-V122	paw swab	IV	Dog	2018	Lab1
BIOS-V123	skin exudate	IV	Dog	2015	Lab1
BIOS-V124	perilabial swab	III	Dog	2015	Lab1
BIOS-V125	skin biopsy	III	Dog	2015	Lab1
BIOS-V127(8)	skin swab	IV	Dog	2016	Lab1
BIOS-V130	Paracostal abscess	I	Rabbit	2003	Lab1
BIOs-V131	finger swab	III	Dog	2014	Lab1
BIOS-V132	chronic wound swab	I	Dog	2015	Lab1
BIOS-V133	skin swab	III	Dog	2014	Lab1
BIOS-V134	interdigital swab	II	Dog	2014	Lab1
BIOS-V135	pustule swab	IV	Dog	2015	Lab1
BIOS-V136	perilabial swab	III	Dog	2015	Lab1
BIOS-V137	skin swab	II	Dog	2014	Lab1
BIOS-V138	paw swab	II	Dog	2018	Lab1
BIOS-V140	skin swab	III	Dog	2015	Lab1
BIOS-V141	skin swab	II	Dog	2015	Lab1
BIOS-V142	skin swab after disinfection	III	Dog	2014	Lab1
BIOS-V143(9)	Hind limb lesion swab	III	Dog	2014	Lab1

The numbers in brackets represent strains collected from the same animal. MSSP: methicillin-susceptible *Staphylococcus pseudintermedius;* MRSP: methicillin-resistant *Staphylococcus pseudintermedius;* MDR: multidrug resistant.

Table S3. Continuation.

Isolate	Biological sample	*agr* type	Host	Year	Lab
BIOS-V144(9)	pustule swab	III	Dog	2014	Lab1
BIOS-V145	ventral pustule	IV	Dog	2018	Lab1
BIOS-V146	skin swab	III	Dog	2015	Lab1
BIOS-V162	nodular lesion swab	IV	Dog	2018	Lab2
BIOS-V164	surgical wound swab	IV	Dog	2018	Lab2
BIOS-V167	submandibular abscess swab	II	Dog	2018	Lab2
BIOS-V170	arm abscess swab	IV	Dog	2018	Lab2
BIOS-V175	skin swab	IV	Dog	2018	Lab2
BIOS-V176	skin swab	II	Dog	2018	Lab2
BIOS-V179	skin swab	III	Dog	2018	Lab2
BIOS-V182	subcutaneous swab	IV	Dog	2018	Lab2
BIOS-V188	hind limb abscess swab	III	Dog	2018	Lab2
BIOS-V189	pyoderma swab	IV	Dog	2018	Lab2
BIOS-V190	papule in armpit	I	Dog	2018	Lab2
BIOS-V194	skin swab	III	Dog	2018	Lab2
BIOS-V195	skin swab	III	Dog	2018	Lab2
BIOS-V196	skin swab	III	Dog	2018	Lab2
BIOS-V207	thigh wound swab	III	Cat	2018	Lab2
BIOS-V211	skin swab	IV	Dog	2018	Lab2
BIOS-V212	interdigital swab	IV	Dog	2018	Lab2
BIOS-V213	amputation thigh material swab	III	Dog	2018	Lab2
BIOS-V214	skin swab	II	Dog	2018	Lab2
BIOS-V217	skin swab	IV	Dog	2018	Lab2
BIOS-V218	skin swab	III	Dog	2018	Lab2
BIOS-V219	skin swab	IV	Dog	2018	Lab2
BIOS-V221	skin swab	I	Dog	2018	Lab2
BIOS-V223	surgical site swab	III	Dog	2018	Lab2
BIOS-V224	face mass swab	II	Dog	2018	Lab2
BIOS-V225	interdigital swab	III	Dog	2018	Lab2
BIOS-V226	pelvic limb swab	II	Dog	2018	Lab2
BIOS-V227	pustule swab	III	Dog	2018	Lab2
BIOS-V228	skin swab	II	Dog	2018	Lab2
BIOS-V230	pelvic limb biopsy	IV	Dog	2018	Lab2
BIOS-V231	skin swab	IV	Dog	2018	Lab2
BIOS-V233	papule swab	IV	Dog	2018	Lab2
BIOS-V234	skin swab	III	Dog	2018	Lab2
BIOS-V235	skin pustule swab	III	Dog	2018	Lab2
BIOS-V236	skin swab	III	Dog	2018	Lab2

The numbers in brackets represent strains collected from the same animal. MSSP: methicillin-susceptible *Staphylococcus pseudintermedius;* MRSP: methicillin-resistant *Staphylococcus pseudintermedius;* MDR: multidrug resistant.

Table S3. Continuation.

Isolate	Biological sample	*agr* type	Host	Year	Lab
BIOS-V237	torso swab	III	Dog	2018	Lab2
BIOS-V238	skin swab	IV	Dog	2018	Lab2
BIOS-V239	limb swab	IV	Dog	2018	Lab2
BIOS-V240	fracture wound swab	III	Cat	2018	Lab2
BIOS-V241	granuloma swab	III	Dog	2018	Lab2
BIOS-V242	biopsy swab	II	Dog	2018	Lab2
BIOS-V244	skin swab	III	Dog	2018	Lab2
BIOS-V247	callus swab	II	Dog	2018	Lab2
BIOS-V249	skin swab	III	Dog	2018	Lab2
BIOS-V251	exudate swab	II	Dog	2018	Lab2
BIOS-V259	skin swab	IV	Dog	2018	Lab2
BIOS-V260	foreskin swab	IV	Dog	2018	Lab2
BIOS-V262	dermal punch	II	Dog	2018	Lab2
BIOS-V263	papule	IV	Dog	2018	Lab2
BIOS-V264	interdigital swab	III	Dog	2018	Lab2
BIOS-V268	skin exudate swab	II	Dog	2018	Lab2
BIOS-V270	skin wound swab	III	Dog	2018	Lab2
BIOS-V273	wound swab	II	Dog	2018	Lab2
BIOS-V276	thigh swab	III	Dog	2018	Lab2
BIOS-V280	surgical material	III	Dog	2018	Lab2
BIOS-V281	skin swab	III	Dog	2018	Lab2
BIOS-V285	skin swab	III	Dog	2018	Lab2
BIOS-V286	pustule swab	III	Dog	2018	Lab2
BIOS-V287	skin swab	III	Dog	2018	Lab2
BIOS-V290	chin swab	III	Dog	2018	Lab2
BIOS-V292	suture swab	II	Cat	2018	Lab2
BIOS-V297	skin swab	III	Dog	2018	Lab2
BIOS-V298	skin swab	I	Dog	2018	Lab2
BIOS-V299	interdigital furunculosis swab	III	Dog	2018	Lab2
BIOS-V302	skin swab	III	Dog	2018	Lab2

The numbers in brackets represent strains collected from the same animal. MSSP: methicillin-susceptible *Staphylococcus pseudintermedius;* MRSP: methicillin-resistant *Staphylococcus pseudintermedius;* MDR: multidrug resistant.

Table S4. Primers used in this study.

Gene	Primers	Sequence (5′-3′)	Amplicon size (pb)	Reference
Staphylococcus pseudintermedius				
lukF	lukF_SP_Fw	CCTGTCTATGCCGCTAATCAA	572	[90]
	lukF_SP_Rv	AGGTCATGGAAGCTATCTCGA		
icaADB	icaADB_SP_Fw	GCAACATGGTCAAGTCCAGA	358	This work
	icaADB_SP_Rv	CGACACACCCTATGGCTATG		
agrD	agrD_SP_Fw	GGGGTATTATTACAATCATTC	300	[88]
	agrD_SP_Rv	CTGATGCGAAAATAAAGGATTG		
Staphylococcus aureus				
lukS-PV/lukF-PV	lukSF_Fw	CATTAGGTAAAATGTCTGG	431	This work
	lukSF_Rv	GCATCAACTGTATTGGAT		
icaADB	icaADB_SA_Fw	AGTTCTTGTCGCATTTCCAA	359	This work
	icaADB_SA_Rv	CACGATTCTCTTCCTCTCTGC		
agr	pan_agr_SA_Fw	ATGCACATGGTGCACATGC		[89]
	agr_I_SA_Rv	GTCACAAGTACTATAAGCTGCGAT	439	
	agr_II_SA_ Rv	TATTACTAATTGAAAAGTGCCATAGC	572	
	agr_III_SA_ Rv	GTAATGTAATAGCTTGTATAATAATAC CCAG	321	
	agr_IV_SA_Rv	CGATAATGCCGTAATACCCG	657	

References

45. Costa, S. S.; Ribeiro, R.; Serrano, M.; Oliveira, K.; Ferreira, C.; Leal, M.; Pomba, C.; Couto, I. *Staphylococcus aureus* causing skin and soft tissues infections in companion animals: antimicrobial resistance profiles and clonal lineages. *Antibiotics* **2022,** 11, 599. https://doi.org/10.3390/antibiotics11050599
46. Morais, C.; Costa, S.S.; Andrade, M.; Ramos, B.; Leal, M.; Abrantes, P.; Pomba, C.; Couto, I. Clonal lineages of *Staphylococcus pseudintermedius* associated with skin and soft tissues infections in pets, Portugal. In Proceedings of the 31st European Congress of Clinical Microbiology and Infectious Diseases, Vienna, Austria, 9–12 July 2021.
47. Costa, S.S.; Oliveira, V.; Serrano, M.; Pomba, C.; Couto, I. Phenotypic and molecular traits of *Staphylococcus coagulans* associated with canine skin infections in Portugal. *Antibiotics* **2021**, *10*, 518. https://doi.org/10.3390/antibiotics10050518
88. Couto, N.; Belas, A.; Oliveira, M.; Almeida, P.; Clemente, C.; Pomba, C. Comparative RNA-seq-Based transcriptome analysis of the virulence characteristics of methicillin-resistant and -susceptible *Staphylococcus pseudintermedius* strains isolated from small animals. *Antimicrob Agents Chemother* **2015**, 60, 962-7. https://doi.org/10.1128/AAC.01907-15
89. Lina, G.; Boutite, F.; Tristan, A.; Bes, M.; Etienne, J.; Vandenesch, F. Bacterial competition for human nasal cavity colonization: role of Staphylococcal *agr* alleles. *Appl Environ Microbiol* **2003**, 69, 18-23. https://doi.org/10.1128/AEM.69.1.18-23.2003
90. Futagawa-Saito, K.; Sugiyama, T.; Karube, S.; Sakurai, N.; Ba-Thein, W.; Fukuyasu, T. Prevalence and characterization of leuko-toxin-producing *Staphylococcus intermedius* in isolates from dogs and pigeons. *J Clin Microbiol* **2004**, 42(11), 5324-6.
